# Wnt/β-catenin signaling contributes to articular cartilage homeostasis through lubricin induction in the superficial zone

**DOI:** 10.1186/s13075-019-2041-5

**Published:** 2019-11-27

**Authors:** Fengjun Xuan, Fumiko Yano, Daisuke Mori, Ryota Chijimatsu, Yuji Maenohara, Hideki Nakamoto, Yoshifumi Mori, Yuma Makii, Takeshi Oichi, Makoto Mark Taketo, Hironori Hojo, Shinsuke Ohba, Ung-il Chung, Sakae Tanaka, Taku Saito

**Affiliations:** 1 Sensory & Motor System Medicine, Graduate School of Medicine, The University of Tokyo, 7-3-1 Hongo, Bunkyo-ku, Tokyo, 113-8655 Japan; 2 Bone and Cartilage Regenerative Medicine, Graduate School of Medicine, The University of Tokyo, 7-3-1 Hongo, Bunkyo-ku, Tokyo, 113-8655 Japan; 3 Division of Oral Anatomy, Department of Human Development and Fostering, Meikai University School of Dentistry, 1-1 Keyakidai, Sakado, Saitama, 350-0283 Japan; 4 Division of Experimental Therapeutics, Graduate School of Medicine, Kyoto University, Yoshida-Konoé-cho, Sakyo, Kyoto, 606-8506 Japan; 5 Center for Disease Biology and Integrative Medicine, Graduate School of Medicine, The University of Tokyo, 7-3-1 Hongo, Bunkyo-ku, Tokyo, 113-8655 Japan

**Keywords:** Osteoarthritis, Chondrocyte, Superficial zone, Wnt signaling

## Abstract

**Background:**

Both loss- and gain-of-function of Wnt/β-catenin signaling in chondrocytes result in exacerbation of osteoarthritis (OA). Here, we examined the activity and roles of Wnt/β-catenin signaling in the superficial zone (SFZ) of articular cartilage.

**Methods:**

Wnt/β-catenin signaling activity was analyzed using TOPGAL mice. We generated *Prg4-Cre*^*ERT2*^*;Ctnnb1*^*fl/fl*^ and *Prg4-Cre*^*ERT2*^*;Ctnnb1-ex3*^*fl/wt*^ mice for loss- and gain-of-function, respectively, of Wnt/β-catenin signaling in the SFZ. Regulation of Prg4 expression by Wnt/β-catenin signaling was examined in vitro, as were upstream and downstream factors of Wnt/β-catenin signaling in SFZ cells.

**Results:**

Wnt/β-catenin signaling activity, as determined by the TOPGAL reporter, was high specifically in the SFZ of mouse adult articular cartilage, where Prg4 is abundantly expressed. In SFZ-specific β-catenin-knockout mice, OA development was significantly accelerated, which was accompanied by decreased Prg4 expression and SFZ destruction. In contrast, Prg4 expression was enhanced and cartilage degeneration was suppressed in SFZ-specific β-catenin-stabilized mice. In primary SFZ cells, Prg4 expression was downregulated by β-catenin knockout, while it was upregulated by β-catenin stabilization by exon 3 deletion or treatment with CHIR99021. Among Wnt ligands, Wnt5a, Wnt5b, and Wnt9a were highly expressed in SFZ cells, and recombinant human WNT5A and WNT5B stimulated Prg4 expression. Mechanical loading upregulated expression of these ligands and further promoted Prg4 transcription. Moreover, mechanical loading and Wnt/β-catenin signaling activation increased mRNA levels of *Creb*1, a potent transcription factor for *Prg4*.

**Conclusions:**

We demonstrated that Wnt/β-catenin signaling regulates Prg4 expression in the SFZ of mouse adult articular cartilage, which plays essential roles in the homeostasis of articular cartilage.

## Introduction

Osteoarthritis (OA), which involves articular cartilage degradation, is the most prevalent joint disorder. A large number of studies using genetically modified mice have revealed signaling pathways or molecules responsible for OA, such as a disintegrin-like metallopeptidase with a thrombospondin type 1 motif 5, hedgehog signaling, syndecan-4, matrix metalloproteinase-13, hypoxia-inducible factor 2-alpha, nuclear factor kappa-light-chain-enhancer of activated B cells, Notch signaling, and Wnt signaling [1–9]. However, the entirety of OA pathophysiology is not yet understood despite all of these findings.

Wnt is one of the most investigated signaling pathways in developmental biology and pathophysiology of various diseases. Wnt genes encode a family of about 20 small secreted proteins, which are highly conserved across various organisms [10]. Wnt receptor activation leads to signal transduction via three different pathways: a canonical Wnt/β-catenin cascade, noncanonical planar cell polarity pathway, and the Wnt/Ca^2+^ pathway [10]. Notably, the Wnt/β-catenin pathway is the best understood of these signaling cascades. When Wnt proteins are not bound to a receptor, a serine/threonine kinase complex that includes Axin, casein kinase 1, and glycogen synthase kinase 3 beta (GSK-3β) phosphorylates β-catenin, which can then be degraded by E3 ubiquitin ligases [10]. However, once Wnt proteins bind their receptors, the kinase complex is recruited to the receptor, which leads to the stabilization of β-catenin [10]. Subsequently, β-catenin translocates into the nucleus, binds to T cell factor/lymphoid enhancer factor (Tcf/Lef), and activates the transcription of target genes [10].

Regulation of chondrocyte generation and differentiation by the canonical Wnt/β-catenin pathway has been extensively investigated using transgenic and tissue-specific knockout mice. Skeletal formation is severely impaired in mouse embryos by chondrocyte-specific constitutive activation of Wnt/β-catenin signaling [11–13]. Interestingly, inactivation or inhibition of Wnt/β-catenin signaling also leads to severe impairment of skeletal growth [14–16]. These findings indicate that Wnt/β-catenin signaling activity is restrictively regulated in chondrocyte generation and differentiation during development [17]. Similarly, regulation of articular cartilage by Wnt/β-catenin signaling is complicated and somewhat contradictory. Transgenic mice expressing an inhibitor of β-catenin and Tcf in chondrocytes displayed enhanced articular cartilage destruction [3]. However, stabilization of β-catenin in adult chondrocytes also enhanced OA progression [7]. Reasons underlying these contradictory findings have not yet been reported.

Recently, roles of articular cartilage superficial zone (SFZ) cells have been the focus of several studies. SFZ cells are thought to have different properties from chondrocytes in the deeper zone (DZ). SFZ cells produce lubricin (encoded by *Prg4*), a protein responsible for the lubrication of articular joints that contributes to the protection of articular cartilage [18, 19]. In the newborn period, joints of *Prg4*-knockout mice appear normal [18]. However, as the mice aged, their joints exhibited early wear and increased friction compared with WT mice [19]. Previous studies have shown that Wnt/β-catenin signaling is constitutively activated in the SFZ and required for SFZ formation during skeletal development [20, 21]. A recent cell tracking study using *Prg4-Cre*^*ERT2*^ mice further revealed that Prg4-expressing cells located at the joint surface in embryos or young mice include a progenitor population for mature DZ chondrocytes [22]. Based on these findings, we hypothesized that Wnt/β-catenin signaling activity may contribute to the maintenance of the SFZ and further homeostasis of articular joints in adulthood similarly to the developmental period.

Herein, we describe roles of Wnt/β-catenin signaling in the SFZ of adult articular cartilage. We examined Wnt/β-catenin signaling activity in the SFZ and its in vivo roles in OA development, using SFZ-specific β-catenin knockout or stabilization. We further investigated expression levels of Wnt ligands and the involvement of mechanical loading as upstream triggers.

## Methods

### Mice

All animal experiments were authorized by the Animal Care and Use Committee of The University of Tokyo. We also complied with all relevant ethical regulations. In each experiment, we compared the genotypes of littermates maintained in a C57BL/6J background. TOPGAL, *Prg4-Cre*^*ERT2*^, *Rosa26-tdTomato* (Ai14), and *Ctnnb1-flox* mice were obtained from The Jackson Laboratory (Bar Harbor, ME) [22–24]. *Ctnnb1-ex3-flox* mice were generated as previously described [25].

### Histological analyses

Tissue samples were fixed in 4% paraformaldehyde buffered with phosphate-buffered saline (PBS, pH 7.4) at 4 °C for 1 day. Specimens were decalcified with 10% EDTA (pH 7.4) at 4 °C for 2 weeks, embedded in paraffin, and 5-μm-thick sagittal sections were cut from specimens. Safranin O staining was performed in accordance with standard protocols. For immunohistochemistry, sections were incubated with antibodies against red fluorescence protein (RFP; 1:2000; 600-401-379, Rockland, Limerick, PA), Ctnnb1 (1:1000, Ab2365, Abcam, Cambridge, UK), and Prg4 (1:500, Ab28484, Abcam). For visualization, simple stain mouse-MAX-PO(R) (Nichirei Bioscience, Tokyo, Japan) was used.

### Osteoarthritis (OA) experiments

For OA experiments, *Prg4-Cre*^*ERT2*^*;Ctnnb1*^*fl/fl*^ and *Prg4-Cre*^*ERT2*^*;Ctnnb1-ex3*^*fl/wt*^ mice were generated by mating *Prg4-Cre*^*ERT2*^ with *Ctnnb1-flox* and *Ctnnb1-ex3-flox* mice, respectively. Tamoxifen (Sigma Aldrich, St. Louis, MO; 100 μg per g of body weight) was intraperitoneally injected into 7-week-old *Prg4-Cre*^*ERT2*^*;Ctnnb1*^*fl/fl*^, *Ctnnb1*^*fl/fl*^, *Prg4-Cre*^*ERT2*^*;Ctnnb1-ex3*^*fl/wt*^, and *Ctnnb1-ex3*^*fl/wt*^ mice daily for 5 days. A surgical procedure was then performed to establish an experimental OA model in 8-week-old male mice [26]. Under general anesthesia, resection of the medial collateral ligament and medial meniscus was performed under a surgical microscope. For sham surgery, only the skin incision was performed. Mice were analyzed 8 weeks after surgery. For the aging model, *Prg4-Cre*^*ERT2*^*;Ctnnb1*^*fl/fl*^ and *Ctnnb1*^*fl/fl*^ mice were analyzed at 18 months of age. All mice were maintained under the same conditions (three mice per cage). OA severity was quantified using the Osteoarthritis Research Society International (OARSI) system [27], which was assessed by two observers blinded to the experimental groups.

### Cell cultures

Primary SFZ cells were isolated as previously described [21]. Briefly, the primary end of the femur and the distal end of the tibia were dissected from P5 mice, and ligaments and tendons were excised. Cartilage tissues were incubated with 0.25% trypsin (Thermo Fisher Scientific, Waltham, MA) for 1 h, followed by 1.5-h digestion with 173 U/mL of type I collagenase (Worthington Biochemical Corporation, Lakewood, NJ). DZ cells were isolated by additional digestion of residual epiphyseal cartilage tissue with 43 U/ml collagenase type I for 5 h. Dissociated cells were seeded on fibronectin-coated culture dishes. Cells were cultured with Dulbecco’s modified Eagle’s medium (DMEM) (Wako, Osaka, Japan) containing 10% fetal bovine serum (FBS). The cells were cultured as a monolayer in all experiments. We used 2 μM 4-hydroxytamoxifen (4OHT; Sigma Aldrich, St Louis, MO, USA) to induce Cre recombination in the cultured SFZ cells. Recombinant human (rh) WNT5A (R&D Systems, Minneapolis, MN), rhWNT5B (R&D Systems), rhWNT9A (R&D Systems), CHIR99021 (Cayman, Ann Arbor, MI), FH535 (Cayman), and 666-15(Tocris Bioscience, Bristol, UK) were used at the concentrations described in the figures and figure legends. For the luciferase assay, ATDC5 cells (Riken BRC, Tsukuba, Japan) were cultured in DMEM/F-12 (Wako) containing 5% FBS.

### Cyclic tensile strain loading of mouse primary SFZ cells

Mouse primary SFZ cells were seeded on silicon stretch chambers at a density of 1 × 10^5^ cells/chamber (chamber area = 2 × 2 cm). The cells were cultured in DMEM containing 10% FBS. Cyclic tensile strain (0.5 Hz, 15% elongation) was applied by an STB-140 mechanical stretch system (STREX, Osaka, Japan) in a CO_2_ incubator. Control cells were cultured without cyclic tensile strain.

### Fluid flow shear stress (FFSS) conditions of mouse primary SFZ cells

Mouse primary SFZ cells were seeded on tissue culture plates (culture plate diameter = 3.5 cm) at a density of 2 × 10^5^ cells/cm^2^. The cells were cultured in DMEM containing 10% FBS. FFSS (180 rotations per minute) was applied by a WB-101SRC orbital shaker (WAKEN, Kyoto, Japan) in a CO_2_ incubator. Control cells were cultured under a static condition.

### Western blotting

Cells were lysed in M-PER mammalian protein extraction reagent (Thermo Fisher Scientific). Cell lysates were fractionated by SDS-PAGE and transferred onto nitrocellulose membranes (Bio-Rad, Hercules, CA). After blocking with 6% skim milk, membranes were incubated with primary antibodies against Ctnnb1 (1:250; #9652, Cell Signaling Technology, Danvers, MA), CREB1 (1:250; 9197S, Cell Signaling Technology), and actin (1:250; AC-74, Sigma Aldrich). Membranes were incubated with a horseradish peroxidase-conjugated antibody (Promega, Madison, WI), and immunoreactive proteins were visualized with ECL prime (GE Healthcare, Chicago, IL) and iBright CL 1000 (Thermo Fisher Scientific). The signal intensity of three independent experiments was quantified using the iBright CL 1000 and normalized to actin.

### RT-qPCR

Total RNA was purified with an RNeasy Mini Kit (Qiagen, Hilden, Germany). One microgram of total RNA was reverse transcribed using ReverTraAce qPCR RT Master Mix with gDNA Remover (Toyobo, Osaka, Japan). Each PCR reaction contained 1× THUNDERBIRD SYBR qPCR Mix (Toyobo), 0.3 mM specific primers, and 20 ng of cDNA. The full-length or partial cDNAs of target genes, including PCR amplicon sequences, were amplified by PCR, cloned into pCR-TOPO Zero II or pCR-TOPO II vectors (Invitrogen), and used as standard templates after linearization. Copy numbers of target mRNAs in each total RNA were calculated by reference to standard curves and adjusted to mouse standard total RNA (Thermo Fisher Scientific) with rodent actin as an internal control. All reactions were run in triplicate. Primer sequences for real-time RT-PCR are shown in Additional file 1: Table S1.

### Luciferase assay

Proximal regions around the transcription start site of *Prg4* (from − 3460, − 1470, or − 500 to + 592 bp relative to the transcription start site) were amplified by PCR using mouse genomic DNA as the template and cloned into the PGL4.10[luc2] vector (Promega). We performed the luciferase assay using ATDC5 cells and a Dual-Luciferase Reporter Assay System (Promega) and reported the data as the ratio of firefly to Renilla activities.

### Statistical analyses

To assess the statistical significance of experimental data, we used a two-tailed unpaired Student’s *t* test for comparison of two groups, one-way ANOVA followed by Tukey’s correction for comparison of multiple groups, and the Mann-Whitney *U* test for the OARSI score, as appropriate. *P* values less than 0.05 were considered significant.

## Results

### Wnt/β-catenin signaling activity in the SFZ

**Fig. 1:**
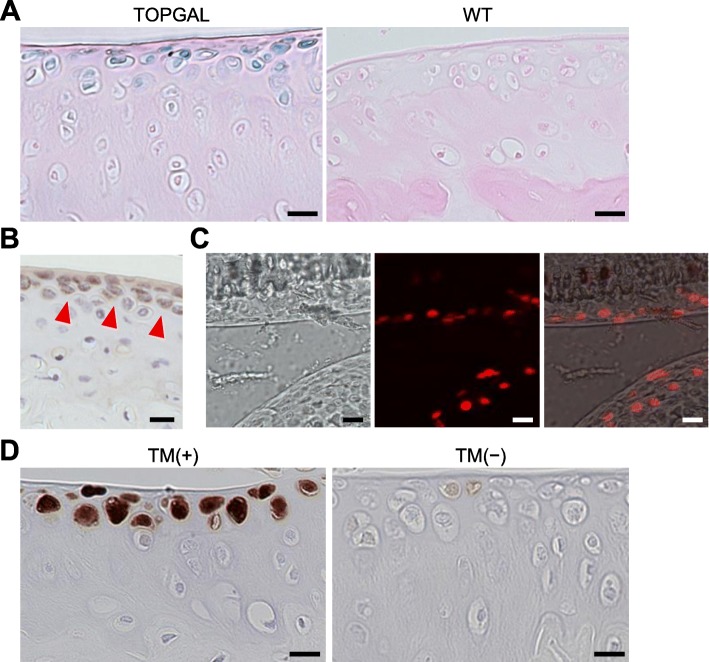
Activity of Wnt/β-catenin signaling in the superficial zone (SFZ) of mouse adult articular cartilage. **a** LacZ staining of tibial articular cartilage of 12-week-old TOPGAL and WT mice. Scale bars, 20 μm. **b** Immunostaining of lubricin (Prg4) in tibial articular cartilage of 12-week-old WT mice. Scale bar, 20 μm. Red arrowheads indicate positive areas. **c** Bright field and fluorescent images of a knee joint of 12-week-old *Prg4-Cre*^*ERT2*^*;Ai14* mouse 3 days after tamoxifen treatment. Scale bars, 20 μm. **d** Immunostaining of red fluorescent protein (RFP) in tibial articular cartilage of 12-week-old *Prg4-Cre*^*ERT2*^*;Ai14* mouse with [TM(+)] or without tamoxifen treatment [TM(−)]. Scale bar, 20 μm

We initially confirmed Wnt/β-catenin signaling activity in articular cartilage of adult mice. In knee joint cartilage of TOPGAL reporter mice, in which a β-galactosidase reporter is driven by β-catenin/Lef1-responsive elements, X-Gal staining was detected in SFZ cells but not chondrocytes of middle and deep zones (Fig. 1a). Prg4, a representative marker protein for the SFZ, was determined to be expressed in a similar area by immunohistochemistry (Fig. 1b). We next examined localization of Cre expression in *Prg4-Cre*^*ERT2*^ mice by mating them with Ai14 mice for further experiments. In frozen sections of *Prg4-Cre*^*ERT2*^;Ai14 knee joint 1 week after tamoxifen induction, fluorescent signal was detected in the SFZ (Fig. 1c). After fixation and paraffin embedding, we confirmed Cre recombination by immunohistochemistry with an antibody against RFP. RFP protein was intensively detected in the SFZ of *Prg4-Cre*^*ERT2*^;Ai14 knee joint after tamoxifen induction, whereas it was scarcely detected in animals without tamoxifen induction (Fig. 1d).

### Enhancement of OA development by β-catenin knockout in the SFZ

**Fig. 2:**
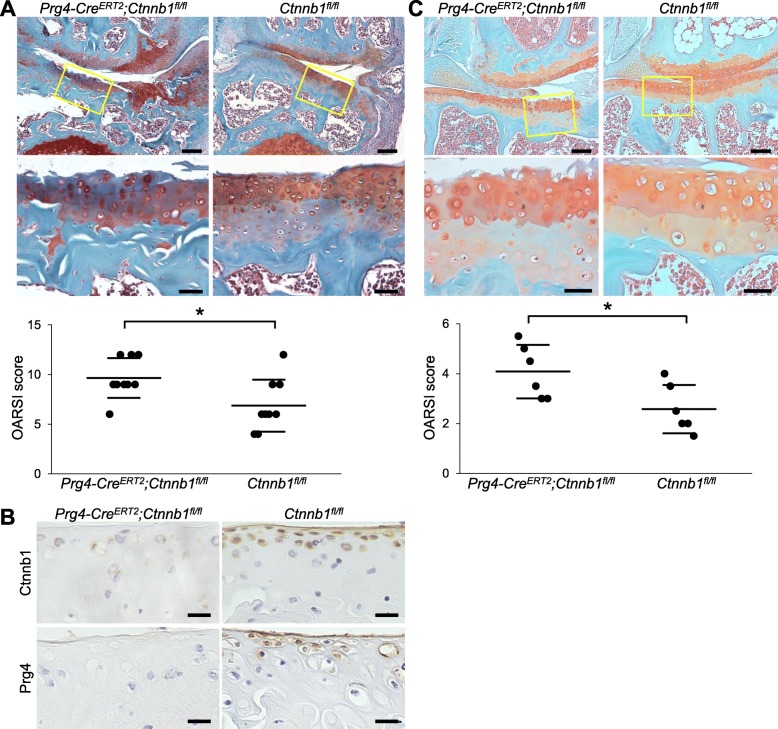
Osteoarthritis development in SFZ-specific *Ctnnb1*-knockout mice. **a** Safranin O staining and OARSI scores of mouse knee joints of *Prg4-Cre*^*ERT2*^*;Ctnnb1*^*fl/fl*^ and *Ctnnb1*^*fl/fl*^ mice 8 weeks after surgery. Tamoxifen induction was performed at 7 weeks. Each experimental group contained nine biologically independent animals. Inset boxes indicate regions of enlarged images below. Scale bars, 50 and 20 μm. *P < 0.05 (Mann-Whitney *U* test). **b** Immunostaining of β-catenin (Ctnnb1) and Prg4 in sham knee joints of *Prg4-Cre*^*ERT2*^*;Ctnnb1*^*fl/fl*^ and *Ctnnb1*^*fl/fl*^ mice 8 weeks after surgery. Scale bars, 20 μm. **c** Safranin O staining and OARSI scores of mouse knee joints of *Prg4-Cre*^*ERT2*^*;Ctnnb1*^*fl/fl*^ and *Ctnnb1*^*fl/fl*^ mice at 18 months. Tamoxifen induction was performed at 7 weeks. Each experimental group contained six biologically independent animals. Inset boxes indicate regions of enlarged images below. Scale bars, 50 and 20 μm. *P < 0.05 (Mann-Whitney *U* test)

We next examined roles of Wnt/β-catenin in SFZ in vivo. To knockout β-catenin in Prg4-expressing cells in a tamoxifen-dependent manner, we generated *Prg4-Cre*^*ERT2*^*;Ctnnb1*^*fl/fl*^ mice by mating *Prg4-Cre*^*ERT2*^ and *Ctnnb1*^*fl/fl*^ mice. We injected tamoxifen into 7-week-old *Prg4-Cre*^*ERT2*^*;Ctnnb1*^*fl/fl*^ and *Ctnnb1*^*fl/fl*^ mice daily for 5 days. At 8 weeks, we created an OA model by surgical destabilization of knee joints [26]. Eight weeks after surgical induction, degradation of articular cartilage was significantly enhanced in *Prg4-Cre*^*ERT2*^*;Ctnnb1*^*fl/fl*^ mice (Fig. 2a), while the sham joints of both genotypes were similar (Additional file 2: Figure S1). Prg4 expression was decreased in the articular cartilage of sham joints in *Prg4-Cre*^*ERT2*^*;Ctnnb1*^*fl/fl*^ mice, as was β-catenin expression (Fig. 2b). We further investigated alterations of articular cartilage with age using these mice. Similar to the results of a surgical model, OA development was significantly accelerated in *Prg4-Cre*^*ERT2*^*;Ctnnb1*^*fl/fl*^ mice at 18 months (Fig. 2c). Notably, the SFZ of *Prg4-Cre*^*ERT2*^*;Ctnnb1*^*fl/fl*^ cartilage was markedly destructed (Fig. 2a, c).

### Suppression of OA development by β-catenin stabilization in the SFZ

**Fig. 3:**
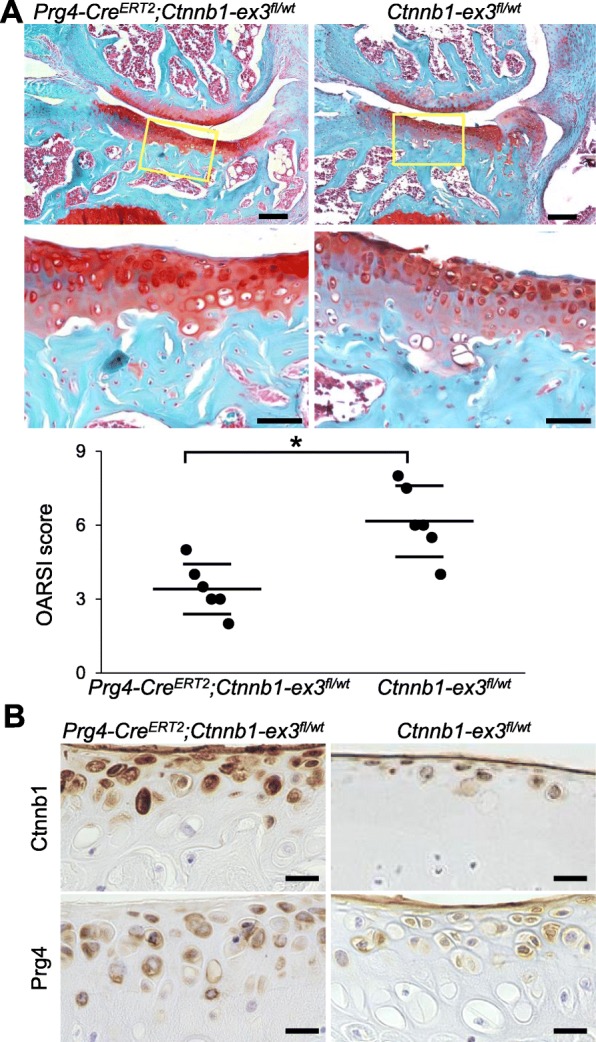
Osteoarthritis development in SFZ-specific β-catenin-stabilized mice. **a** Safranin O staining and OARSI scores of mouse knee joints of *Prg4-Cre*^*ERT2*^*;Ctnnb1-ex3*^*fl/wt*^ and *Ctnnb1-ex3*^*fl/wt*^ mice 8 weeks after surgery. Tamoxifen induction was performed at 7 weeks. Each experimental group contained six biologically independent animals. Inset boxes indicate regions of enlarged images below. Scale bars, 50 and 20 μm. *P < 0.05 (Mann-Whitney *U* test). **b** Immunostaining of Ctnnb1 and Prg4 in sham knee joints of *Prg4-Cre*^*ERT2*^*;Ctnnb1-ex3*^*fl/wt*^ and *Ctnnb1-ex3*^*fl/wt*^ mice 8 weeks after surgery. Scale bars, 20 μm

To examine gain-of-function, we prepared SFZ-specific β-catenin-stabilized mice by mating *Prg4-Cre*^*ERT2*^ and *Ctnnb1-ex3*^*fl/wt*^ mice. Deletion of exon 3 of the *Ctnnb1* gene results in stabilization of β-catenin protein, as it becomes resistant to phosphorylation by GSK-3β [25]. We injected tamoxifen into 7-week-old *Prg4-Cre*^*ERT2*^*;Ctnnb1-ex3*^*fl/wt*^ and *Ctnnb1-ex3*^*fl/wt*^ littermates daily for 5 days and created a surgical OA model. At 8 weeks after surgical induction, OA development was significantly suppressed in *Prg4-Cre*^*ERT2*^*;Ctnnb1-ex3*^*fl/wt*^ mice (Fig. 3a). In addition, destruction of the SFZ was relatively suppressed in *Prg4-Cre*^*ERT2*^*;Ctnnb1-ex3*^*fl/wt*^ cartilage, although it was commonly observed in control cartilage (Fig. 3a). The sham joints of both genotypes were similar (Additional file 2: Figure S1). β-catenin and Prg4 were both upregulated by the deletion of exon 3 of *Ctnnb1* (Fig. 3b). Collectively, these in vivo data suggest that Wnt/β-catenin signaling in the SFZ contributes to articular cartilage homeostasis.

### Regulation of Prg4 expression by Wnt/β-catenin signaling in the SFZ

**Fig. 4:**
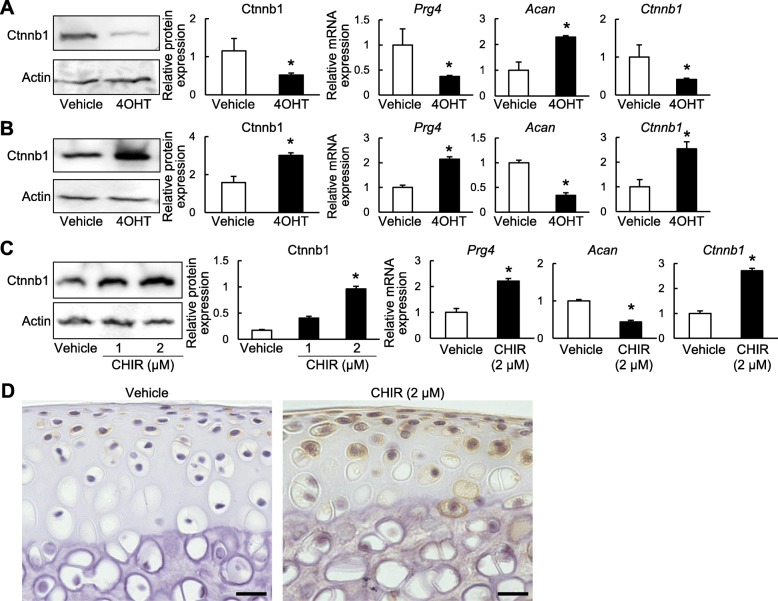
Regulation of *Prg4* expression by Wnt/β-catenin signaling. **a**–**c** Protein levels of Ctnbn1 and mRNA levels of *Prg4*, *Acan*, and *Ctnnb1* in SFZ cells derived from **a** *Prg4-Cre*^*ERT2*^*;Ctnnb1*^*fl/fl*^ mice treated with 4-hydroxytamoxifen (4OHT) or vehicle for 48 h, **b** *Prg4-Cre*^*ERT2*^*;Ctnnb1-ex3*^*fl/wt*^ mice treated with 4OHT or vehicle for 48 h, or **c** WT SFZ cells treated with CHIR99021 or vehicle for 48 h. **d** Immunostaining of Prg4 in mouse femoral heads cultured with CHIR99021 or vehicle for 48 h. Scale bars, 20 μm. All data are expressed as mean ± SD of three biologically independent samples per group. *P < 0.05 versus vehicle (Student’s unpaired two-tailed *t* test)

We next examined the effects of Wnt/β-catenin signaling in the SFZ with in vitro experiments using primary cells. We first confirmed appropriate isolation of SFZ cells by quantification of marker gene expression (Additional file 3: Figure S2a, b). We then isolated SFZ cells from *Prg4-Cre*^*ERT2*^*;Ctnnb1*^*fl/fl*^ and *Prg4-Cre*^*ERT2*^*;Ctnnb1-ex3*^*fl/wt*^ mice as previously described [21] and treated the cells with 4OHT to induce Cre recombination. Western blotting and RT-qPCR confirmed downregulation of β-catenin in 4OHT-treated *Prg4-Cre*^*ERT2*^*;Ctnnb1*^*fl/fl*^ cells (Fig. 4a). Moreover, mRNA levels of *Prg4* were significantly decreased by β-catenin knockdown, while expression of aggrecan (*Acan*), a representative matrix gene in mature chondrocytes, was upregulated (Fig. 4a). In contrast, β-catenin expression was upregulated by 4OHT in *Prg4-Cre*^*ERT2*^*;Ctnnb1-ex3*^*fl/wt*^ cells (Fig. 4b), which exhibited significantly increased *Prg4* expression and β-catenin stabilization and decreased *Acan* expression (Fig. 4b). We next treated SFZ cells from WT mice with CHIR99021, a GSK-3β inhibitor that is widely used as an activator of Wnt/β-catenin signaling. *Prg4* was upregulated and *Acan* was downregulated by CHIR99021 treatment, which was accompanied by increased β-catenin (Fig. 4c); this effect was also observed in 4OHT-treated *Prg4-Cre*^*ERT2*^*;Ctnnb1-ex3*^*fl/wt*^ cells (Fig. 4b). In addition, when we applied CHIR99021 to organ culture of mouse femoral heads, Prg4 expression was intensively enhanced (Fig. 4d).

### Activation of Wnt/β-catenin signaling by mechanical loading

**Fig. 5:**
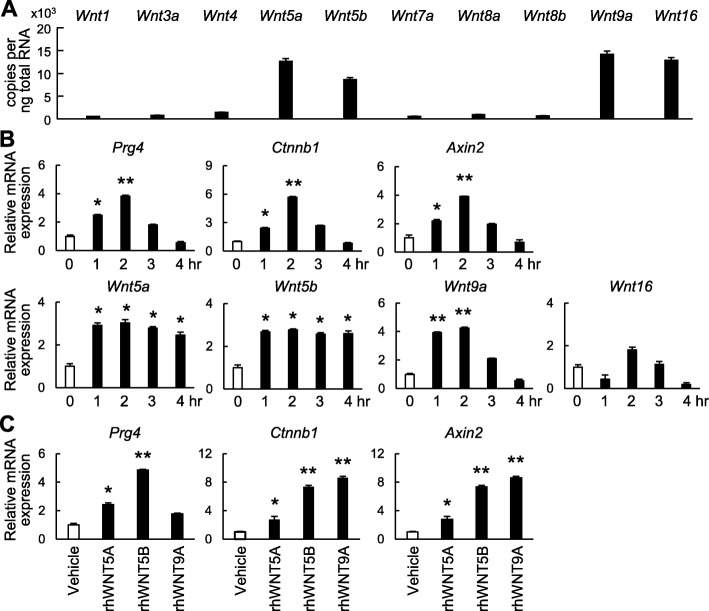
Triggers of Wnt/β-catenin signaling in SFZ cells. **a** mRNA levels of Wnt ligands in SFZ cells. **b** mRNA levels of *Prg4*, *Ctnnb1*, *Axin2*, and Wnt ligands in SFZ cells loaded with cyclic tensile strain. **c** mRNA levels of *Prg4*, *Ctnnb1*, and *Axin2* in SFZ cells treated with 100 ng/ml recombinant human (rh) WNT5A, WNT5B, WNT9A, or vehicle for 1 week. All data are expressed as mean ± SD of three biologically independent samples per group. *P < 0.05 and **P < 0.01 versus vehicle or 0 h (one-way ANOVA followed by Tukey’s correction)

We further examined triggers of Wnt/β-catenin signaling activation in the SFZ. First, mRNA levels of representative Wnt ligands were examined in SFZ cells. *Wnt5a*, *Wnt5b*, and *Wnt9a* were abundantly expressed in SFZ cells, while expression levels of other Wnt ligands were low (Fig. 5a). We next examined the effects of mechanical loading, as Prg4 induction and Wnt/β-catenin signaling activity are affected by mechanical loading [28, 29]. We applied cyclic tensile strain to SFZ cells for 0–4 h. mRNA levels of *Prg4*, *Ctnnb1*, and *Axin2* began to increase at 1 h, peaked at 2 h, and decreased thereafter (Fig. 5b). mRNA levels of *Wnt5a* and *Wnt5b* were upregulated from 1 to 4 h, while *Wnt9a* was increased from 1 to 2 h (Fig. 5b). Alteration of *Wnt16* expression was irregular throughout (Fig. 5b). These data indicate that mechanical loading is one trigger that stimulates Wnt/β-catenin signaling, probably via induction of Wnt ligands. Treatment with rhWNT5A, rhWNT5B, or rhWNT9A enhanced *Ctnnb1* and *Axin2* expression in SFZ cells (Fig. 5c). However, Prg4 expression was significantly increased by rhWNT5A and rhWNT5B, but not by rhWNT9A (Fig. 5c).

### CREB1 induction by Wnt/β-catenin signaling

**Fig. 6:**
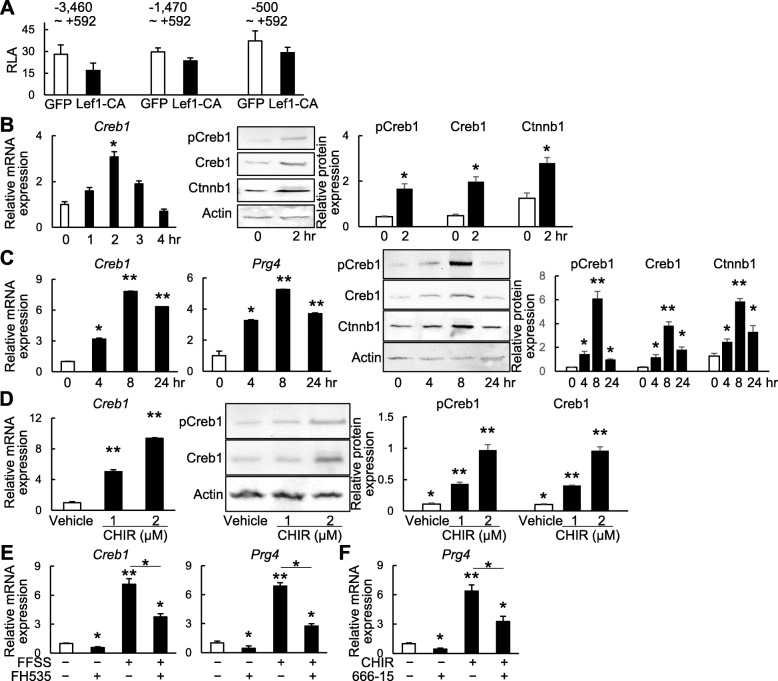
*Prg4* induction by Wnt/β-catenin signaling via CREB1. **a** Luciferase activity of ATDC5 cells transfected with one of three different reporter vectors containing proximal regions around the transcription start site of *Prg4*, and co-transfected with constitutively active Lef1 (Lef1-CA) or GFP control. RLA, relative luciferase activity. *P < 0.05 (Student’s unpaired two-tailed *t* test). **b** mRNA and protein levels of CREB1 in SFZ cells loaded with cyclic tensile strain. *P < 0.05 versus 0 h (one-way ANOVA followed by Tukey’s correction). **c** mRNA levels of CREB1 and Prg4 and protein levels of Ctnnb1, CREB1, and phosphorylated CREB1 (pCREB1) in SFZ cells loaded with fluid flow shear stress (FFSS). *P < 0.05 and **P < 0.01 versus 0 h (one-way ANOVA followed by Tukey’s correction). **d** mRNA levels of CREB1 and protein levels of CREB1 and pCREB1 in SFZ cells treated with CHIR9021 for 48 h. *P < 0.05 and **P < 0.01 versus (one-way ANOVA followed by Tukey’s correction). **e** mRNA levels of CREB1 and Prg4 in SFZ cells after 8-h FFSS loading with or without 15 μM FH535 treatment. FH535 was added to the medium at 2 h before FFSS loading. *P < 0.05 and **P < 0.01 versus 0 h (one-way ANOVA followed by Tukey’s correction). **f** mRNA level of Prg4 in SFZ treated with or without 2 μM CHIR99021 and 100 nM 666-15 for 12 h. *P < 0.05 and **P < 0.01 versus 0 h (one-way ANOVA followed by Tukey’s correction). All data are expressed as mean ± SD of three biologically independent samples per group

Finally, we investigated one potential mechanism underlying Prg4 induction by Wnt/β-catenin signaling. To examine whether *Prg4* is a direct transcriptional target of the Lef-β-catenin complex, we performed a luciferase assay using three reporter vectors containing proximal regions around the transcription start site of *Prg4*. Overexpression of a constitutively active Lef1 (Lef1-CA) did not enhance transactivation of any *Prg4* reporter (Fig. 6a). We next examined CREB1, which is already known as a potent upstream transcription factor of the *Prg4* gene [28]. Notably, *Creb1* expression was increased by cyclic tensile strain (Fig. 6b) in a similar pattern to *Prg4* (Fig. 5c). CREB1 protein was also increased 2 h after stress loading, as was β-catenin (Fig. 6b). Expression of CREB1 and Prg4 was also increased by FFSS in a similar pattern (Fig. 6c). Moreover, CREB1 expression was enhanced by CHIR99021 treatment at both mRNA and protein levels (Fig. 6d). The increase of CREB1 and Prg4 induced by FFSS was partially, but significantly, decreased by the Wnt/β-catenin signaling inhibitor FH535 (Fig. 6e). The Prg4 induction by CHIR99021 was also significantly attenuated by the CREB1 inhibitor 666-15 (Fig. 6f). These results suggest that CREB1 is associated with Prg4 induction by Wnt/β-catenin signaling.

## Discussion

The present study demonstrated that Wnt/β-catenin signaling plays essential roles in the SFZ of adult articular cartilage. Indeed, Wnt/β-catenin signaling is activated only in the SFZ of adult articular cartilage. Loss-of-function in Prg4-positive cells leads to enhanced OA development with degenerated SFZ, while gain-of-function results in resistance to OA. Prg4 expression is dependent on Wnt/β-catenin signaling, and mechanical stress loading increases expression of Wnt ligands and Prg4 in SFZ cells. Wnt/β-catenin signaling induces CREB1, a representative transcription factor of *Prg4*. Similar to previous findings that Wnt/β-catenin signaling contributes to characterization and formation of the SFZ during skeletal development [21], it is also necessary for the maintenance of adult articular cartilage.

Previous studies have shown that activation of Wnt/β-catenin signaling in chondrocytes enhances endochondral ossification and replacement by bone [14, 30]. These findings are consistent with a previous report of enhanced OA progression in *Col2a1-Cre*^*ERT2*^*;Ctnnb1-ex3*^*fllwt*^ mice [7]. In this previous study, Safranin O and Alcian blue staining was reduced, and the articular cartilage area was reduced significantly in *Col2a1-Cre*^*ERT2*^*;Ctnnb1-ex3*^*fllwt*^ mice without surgical OA induction at 2 months after tamoxifen injection [7]. However, exacerbation of OA was also observed in transgenic mice expressing an inhibitor of β-catenin and Lef1 (ICAT) under the *Col2a1* promoter [3]. The cartilage degeneration in transgenic mice was rather slow and mild, which may be partially because the binding of ICAT to β-catenin was not irreversible [3]. Notably, in *Col2a1-ICAT* mice, mild cartilage degeneration was observed at the articular surface, particularly in the weight-bearing area of knee joints [3]. Considering that *Col2a1* is expressed both in the SFZ and DZ, exacerbation of OA in *Col2a1-ICAT* mice may be associated with decreased activity of Wnt/β-catenin signaling in the SFZ, although Prg4 expression was not examined. In the present study, *Prg4-Cre*-driven β-catenin stabilization led to resistance against OA accompanied by enhanced expression of Prg4, whereas *Prg4-Cre*-driven β-catenin knockout resulted in OA exacerbation, indicating that Wnt/β-catenin signaling in the SFZ contributes to cartilage homeostasis at least by Prg4 induction. A recent study showed that SFZ cells include a population of progenitors for articular chondrocytes [22]. Considering that Wnt is a major regulator of mesodermal cells [31], Wnt/β-catenin signaling may be involved not only in *Prg4* induction, but in maintenance of chondrocyte progenitors in the SFZ of adult articular cartilage.

The mechanisms underlying the high expression level of β-catenin protein in the SFZ are still unknown. We found that Wnt5a, Wnt5b, Wnt9a, and Wnt16 were highly expressed in SFZ cells (Fig. 5a). Among them, mRNA expression of Wnt5a, Wnt5b, and Wnt9a was induced by mechanical stress loading (Fig. 5b). However, we could not obtain further findings to explain the upregulation of Wnt/β-catenin signaling in the SFZ. Notably, a recent study showed that Wnt16 protects articular cartilage by antagonization of excessive Wnt/β-catenin signaling [32]. Wnt16 expression is increased in the SFZ during the early stage of mouse experimental knee OA [32]. Treatment with recombinant Wnt16 induces Prg4 expression, although the molecular pathway is unknown [32]. It is widely known that the potent Wnt antagonist FRZB is expressed in DZ chondrocytes and exerts cartilage-protective effects [33, 34]. Various molecules are likely to be involved in the tight regulation of Wnt/β-catenin signaling in the SFZ.

CREB, a member of the leucine zipper family of DNA binding proteins, was identified as a protein that binds to the cAMP-responsive element [35]. Generally, transcriptional activity of CREB is regulated by phosphorylation [36]. Notably, in the present study, *Creb1* mRNA expression was induced by Wnt/β-catenin signaling. Although the interaction of Wnt/β-catenin signaling and CREB-binding protein has been well described [37], induction of CREB by Wnt/β-catenin signaling has not been revealed. However, as both CREB and Wnt/β-catenin are widely expressed, the Wnt/β-catenin-CREB pathway may be involved in other cell activities. We could not reveal the precise molecular mechanisms underlying transcriptional induction of CREB by Wnt/β-catenin, i.e., whether the induction is direct or indirect. Furthermore, molecules or signaling pathways other than CREB may be also associated with Prg4 induction by Wnt/β-catenin signaling.

Ogawa et al. previously reported that mechanical loading enhances *Prg4* expression both in vivo and in vitro [28]. Their results indicated that FFSS induces Prg4 mainly via protein kinase A (PKA)/CREB signaling [28]. Enhanced prostaglandin E2 production and parathyroid hormone-related protein expression elicited CREB phosphorylation through PKA, resulting in increased Prg4 expression [28]. In their study, FFSS enhanced the activity of a luciferase reporter containing TCF-binding sites (TOP-luc), as well as CRE-luc [28]. Considering the present data, both enhanced CREB transcription induced by Wnt/β-catenin and CREB phosphorylation induced by the PKA pathway are likely involved in Prg4 induction by mechanical loading.

## Conclusions

In conclusion, we demonstrated that Wnt/β-catenin signaling is activated specifically in the SFZ of adult mouse articular cartilage, whereby it contributes to *Prg4* expression, which plays essential roles in the homeostasis of articular cartilage.

## Supplementary information

**Additional file 1: Table S1.** Primers used for RT-qPCR.

**Additional file 2: Figure S1.** RepresentativeSafranin O staining of sham knee joints in (a) *Prg4-CreERT2;Ctnnb1*^*fl/fl*^, *Ctnnb1*^*fl/fl*^, (b) *Prg4-CreERT2;Ctnnb1-ex3*^*fl/wt*^, and *Ctnnb1-ex3*^*fl/wt*^mice at 8 weeks post-surgery. Tamoxifen induction was performed at 7 weeks. Insets indicate regions of enlarged images below. Scale bars, 50 and 20 μm.

**Additional file 3: Figure S2.** Validation of primary SFZ cells isolated from joints of P5 mice. a Morphology of SFZ cells and deeper zone (DZ) chondrocytes after 1 week of culture. Scale bars, 50 μm. b mRNA levels of marker genes in SFZ and DZ cells. *P < 0.05 versus vehicle (Student’s unpaired two-tailed t-test).

## Data Availability

The datasets analyzed during the current study are available from the corresponding author on reasonable request.

## Abbreviations

OA: Osteoarthritis
SFZ: Superficial zone
GSK-3β: Glycogen synthase kinase 3 beta
Tcf/Lef: T cell factor/lymphoid enhancer factor
DZ: Deeper zone
PBS: Phosphate-buffered saline
RFP: Red fluorescence protein
OARSI: Osteoarthritis Research Society International
4OHT: 4-Hydroxytamoxifen
rh: Recombinant human
Lef1-CA: Constitutively active Lef1
FFSS: Fluid flow shear stress
PKA: Protein kinase A
