# Supplementary material for: Wnt/β-catenin signaling contributes to articular cartilage homeostasis through lubricin induction in the superficial zone
Source: Arthritis Res Ther. 2019 Nov 27;21:247. doi: 10.1186/s13075-019-2041-5 (PMC6880374; doi:10.1186/s13075-019-2041-5)
Supplement: Supplementary file 1 — Additional file 1: Table S1. Primers used for RT-qPCR. [file 13075_2019_2041_MOESM1_ESM.pdf]

Supplementary Table 1 Primers used for RT-qPCR

Gene Symbol		Sequence	Gene Symbol		Sequence
Wnt1	F	ccgagaaacagcgttcatct	*Wnt16*	F	gccactaccacttccaccc
	R	ggttcatgaggaagcgtagg		R	gagccaccattctgcaagg
Wnt3a	F	caccaccgtcagcaacagcc	*Ctnnb1*	F	gtgcaattcctgagctgaca
	R	aggagcgtgtcactgcgaaag		R	cttaaagatggccagcaagc
Wnt4	F	aacggaaccttgaggtgatg	*Prg4*	F	caagaagcccacctctacca
	R	tcacagccacacttctccag		R	cacctccatctgcatcttca
Wnt5a	F	caaataggcagccgagagac	*Creb1*	F	tcagccgggtactaccattc
	R	ctctagcgtccacgaactcc		R	ctctcttccgtgctgcttct
Wnt5b	F	tggaccccagtcctgactac	*Acan*	F	ccaaaccagcctgacaactt
	R	aaagcaacaccagtggaacc		R	tctagcatgctccaccactg
Wnt7a	F	cgagagctaggctacgtgct	*Axin2*	F	gagtagcgccgtgttagtgact
	R	ctgaggggctgtcttattgc		R	ccaggaaagtccggaagaggtatg
Wnt8a	F	ccatcatgtacgcagtcacc	*Col2a1*	F	gccaagacctgaaactctgc
	R	gccctgttgttgtgaaggtt		R	gccatagctgaagtggaagc
Wnt8b	F	gtggacttcgaagcgctaac	*Erg*	F	ccagcgtcctcagttagatccttacca
	R	ctgcttggaaattgcctctc		R	tcatgttgggcttgctcttcctctc
Wnt9a	F	tgctttcctctacgccatct	*Actin*	F	agatgtggatcagcaagcag
	R	cccaggaactccttgacaaa		R	gcgcaagttaggttttgtca
