# Supplementary material for: Wnt/β-catenin signaling contributes to articular cartilage homeostasis through lubricin induction in the superficial zone
Source: Arthritis Res Ther. 2019 Nov 27;21:247. doi: 10.1186/s13075-019-2041-5 (PMC6880374; doi:10.1186/s13075-019-2041-5)
Supplement: Supplementary file 2 — Additional file 2: Figure S1. RepresentativeSafranin O staining of sham knee joints in (a) Prg4-CreERT2;Ctnnb1fl/fl, Ctnnb1fl/fl, (b) Prg4-CreERT2;Ctnnb1-ex3fl/wt, and Ctnnb1-ex3fl/wtmice at 8 weeks post-surgery. Tamoxifen induction was performed at 7 weeks. Insets indicate regions of enlarged images below. Scale bars, 50 and 20 μm. [file 13075_2019_2041_MOESM2_ESM.pdf]

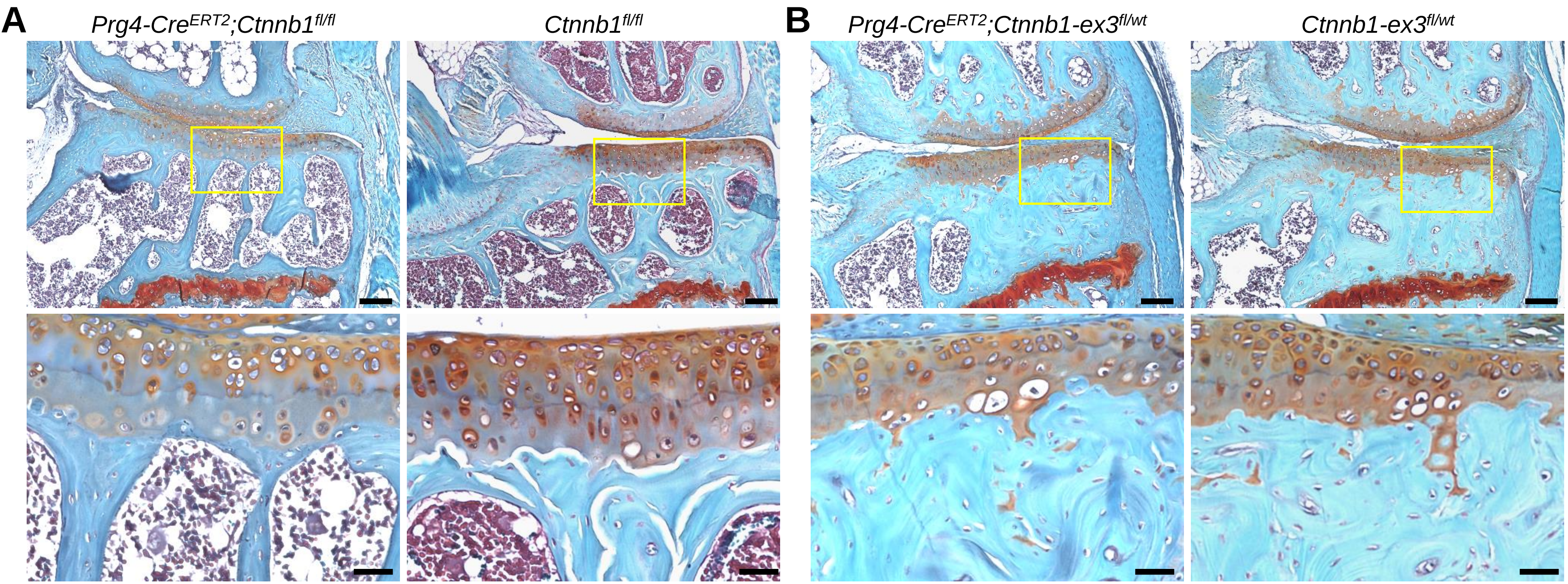

Fig. S1 Representative Safranin O staining of sham knee joints in (**a**) *Prg4-Cre*^{ERT2}*;Ctnnb1*$^{fl/fl}$, *Ctnnb1*$^{fl/fl}$, (**b**) *Prg4-Cre*^{ERT2}*;Ctnnb1-ex3*$^{fl/wt}$, and *Ctnnb1-ex3*$^{fl/wt}$ mice at 8 weeks post-surgery. Tamoxifen induction was performed at 7 weeks. Insets indicate regions of enlarged images below. Scale bars, 50 and 20 μm.
