# Supplementary material for: Wnt/β-catenin signaling contributes to articular cartilage homeostasis through lubricin induction in the superficial zone
Source: Arthritis Res Ther. 2019 Nov 27;21:247. doi: 10.1186/s13075-019-2041-5 (PMC6880374; doi:10.1186/s13075-019-2041-5)
Supplement: Supplementary file 3 — Additional file 3: Figure S2. Validation of primary SFZ cells isolated from joints of P5 mice. a Morphology of SFZ cells and deeper zone (DZ) chondrocytes after 1 week of culture. Scale bars, 50 μm. b mRNA levels of marker genes in SFZ and DZ cells. *P < 0.05 versus vehicle (Student’s unpaired two-tailed t-test). [file 13075_2019_2041_MOESM3_ESM.pdf]

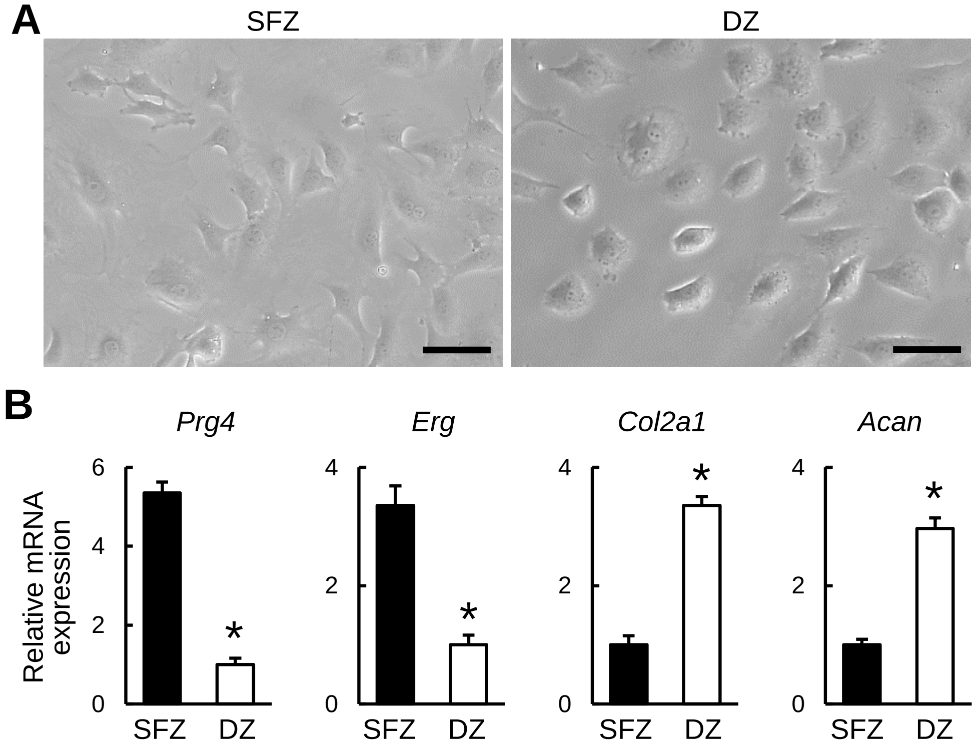

Fig. S2 Validation of primary SFZ cells isolated from joints of P5 mice. **a** Morphology of SFZ cells and deeper zone (DZ) chondrocytes after 1 week of culture. Scale bars, 50 µm. **b** mRNA levels of marker genes in SFZ and DZ cells. **P* < 0.05 versus vehicle (Student's unpaired two-tailed t-test).
